# Health Profiles of Inmates: A Cross-Sectional Study of Prevalent Diseases in a Central Italian Prison

**DOI:** 10.3390/healthcare13172090

**Published:** 2025-08-22

**Authors:** Massimo Lancia, Luca Tomassini, Roberto Scendoni, Elisa Fanella, Alessio Gili, Angela Gambelunghe, Mauro Bacci, Kyriaki Aroni, Virginia Goracci, Cristiana Gambelunghe

**Affiliations:** 1 Forensic Medicine, Forensic Science and Sports Medicine Section, Department of Medicine and Surgery, University of Perugia, 06132 Perugia, Italy; massimo.lancia@unipg.it (M.L.); maurobacci@live.it (M.B.); kyriaki.aroni@collaboratori.unipg.it (K.A.); virginia.goracci@collaboratori.unipg.it (V.G.); cristiana.gambelunghe@unipg.it (C.G.); 2 School of Advanced Studies, University of Camerino, 62032 Camerino, Italy; 3 Institute of Legal Medicine, Department of Law, University of Macerata, 62100 Macerata, Italy; r.scendoni@unimc.it; 4 Department of Internal Medicine, Bruneck Hospital, Azienda Sanitaria dell’Alto Adige, 39100 Bruneck, Italy; elisa.fanella@sabes.it; 5 Department of Life Sciences, Health, and Healthcare Professions, Link Campus University, 00165 Rome, Italy; a.gilli@unilink.it; 6 Department of Medical and Surgical Sciences, Alma Mater Studiorum-University of Bologna, 40126 Bologna, Italy; angela.gambelunghe@unibo.it

**Keywords:** prison health systems, risk factors for health, inmates’ health, inmate and mental illness, constitutional rights

## Abstract

**Background:** Article 32 of the Italian Constitution guarantees the right to health for all citizens, including detainees. Prison populations face unique health challenges due to high-risk lifestyles, psychosocial stressors, and limited access to care. This study aimed to investigate the burden of chronic diseases and associated risk factors among male inmates in a central Italian prison. **Methods:** This cross-sectional study was conducted in accordance with STROBE guidelines at Giuseppe Pagliei Prison in Frosinone, Central Italy, from May 2022 to May 2023. A total of 477 adult male inmates underwent systematic clinical evaluations and medical record reviews. Demographic and health data were analyzed to determine the prevalence of chronic conditions and related risk factors. **Results:** Participants (mean age 47.3 ± 13.1 years; 69.6% Italian, 30.4% international, mainly Eastern European and African) presented on average 1.8 chronic conditions. The most frequent diagnoses were psychiatric disorders (19.9%), cardiovascular diseases (17.2%), and osteoarticular disorders (14.5%). Disease burden correlated with aging, unhealthy lifestyles, and incarceration-related stressors. Tobacco smoking was highly prevalent. **Conclusions:** Male inmates show a considerable and partly preventable burden of chronic disease. Broader policy measures, including alternative sentencing and community-based rehabilitation, may mitigate the health impact of imprisonment while ensuring public safety. Adequate prison healthcare remains a public health priority and a constitutional and human rights obligation.

## 1. Introduction

In Italy, the fundamental right to health is enshrined in Article 32 of the Constitution, which guarantees medical care to all individuals, including those deprived of liberty [1,2]. This right encompasses not only the provision of general and specialized care, but also public health measures aimed at preserving individual well-being [3]. At the international level, both the World Health Organization (WHO) and the Charter of Fundamental Rights of the European Union reaffirm that persons deprived of liberty must receive adequate medical assistance and hygienic conditions [4,5].

In Italy, since 2008 [6], the responsibilities for general and specialist healthcare within penitentiary facilities, as well as employment relations and financial and instrumental resources, have belonged to the National Health Service (having been transferred from the penitentiary administration), and subsequently to the Regions and Local Health Authorities.

This structural transition was designed to ensure care equivalence between detained individuals and the general population. However, its implementation has revealed significant criticalities, such as regional heterogeneity in service provision, limited integration between prison health services and community healthcare, and persistent barriers to accessing preventive, psychiatric, and addiction services [7]. To date, no systematic national evaluation has been conducted to assess the impact or effectiveness of this reform in carceral settings.

These shortcomings are particularly concerning given that inmates are at heightened risk of developing both chronic and infectious diseases due to multiple factors, including pre-existing health conditions, substance abuse, mental health disorders, and environmental stressors within correctional facilities [7,8]. The prison setting presents a unique epidemiological landscape, where psychiatric disorders, cardiovascular conditions, and communicable diseases are particularly prevalent. Studies indicate that incarcerated individuals experience higher rates of mental illness and substance dependence than the general population, with limited access to appropriate healthcare interventions [9,10,11]. Additionally, smoking and poor dietary habits contribute to the exacerbation of metabolic and cardiovascular conditions among detainees [12,13].

While overcrowding has been identified as a challenge in some Italian prisons, it is not uniformly severe across all facilities [13]. As of 28 February 2025, the national prison population stood at 62,165 inmates for a total regulatory capacity of 51,323 places, corresponding to an overall occupancy rate of 121.1% [14]. However, this rate is not evenly distributed across all institutions, and its impact on inmate health varies accordingly. In the case of Frosinone Prison, the facility operates at 99.8% occupancy, which is below the national average [15]. Thus, while overcrowding remains a concern in some locations, the primary focus of this study is on broader inmate health issues rather than the direct consequences of overpopulation.

The health conditions of people living in prison in Italy present significant challenges, with a high prevalence of psychiatric disorders (41.3%), digestive system diseases (14.5%), infectious diseases (11.5%), and cardiovascular conditions (11.4%) compared to the general population [13]. The high rate of psychological distress in correctional facilities has been attributed to both the conditions of incarceration and pre-existing factors, with studies highlighting a considerable incidence of substance dependence and self-harming behaviors, particularly among female inmates [16,17]. Moreover, physical inactivity and limited access to structured exercise programs further compromise the quality of life of people living in prison, negatively impacting both their physical and mental health [18]. Although the 2008 reform transferred the management of prison healthcare to the National Health Service, persistent challenges remain in ensuring continuity of care and access to specialized medical services, exacerbated by organizational inefficiencies and disparities among different penitentiary facilities [18,19].

This study provides a descriptive analysis of inmate health at Giuseppe Pagliei Prison in Frosinone, Central Italy, during the period from May 2022 to May 2023. Its primary objective is to identify the most prevalent diseases and their associated risk factors within the incarcerated population. By collecting and analyzing clinical data, the study assesses the burden of psychiatric disorders, chronic illnesses, infectious diseases, and substance use among inmates, while also evaluating the impact of lifestyle factors, such as smoking, diet, and physical inactivity, on health status. By offering a comprehensive health profile within a single facility, the study aims to contribute to a deeper understanding of the complex interplay between detention and health. In doing so, it underscores the importance of ensuring not only medical care, but also the protection of inmates’ dignity and fundamental rights, in accordance with constitutional and international principles.

## 2. Materials and Methods

This cross-sectional descriptive study, conducted in accordance with the STROBE (Strengthening the Reporting of Observational Studies in Epidemiology) guidelines, analyzed the health records of all adult male inmates (N = 477) at Giuseppe Pagliei Prison, Frosinone, Central Italy, between May 2022 and May 2023. All included subjects had complete demographic, clinical, and lifestyle data.

### 2.1. Participants and Inclusion/Exclusion Criteria

The study population included all male inmates present during the study period who had complete medical records and provided consent for data collection and analysis.

### 2.2. Data Sources and Variables

Health information was extracted from official prison clinical records, including initial medical assessments and follow-up visits. Diagnoses were classified using the International Classification of Diseases, Tenth Revision, Clinical Modification (ICD-10-CM). Variables collected included chronic medical conditions, age, country of origin, and tobacco use, self-reported by inmates, including the number of cigarettes smoked per day. Information on prescribed medications, including psychotropic drugs, was obtained exclusively from medical records, ensuring that only clinically prescribed treatments were considered in the analysis.

### 2.3. Standardization and Data Quality

To ensure diagnostic reliability and consistency, only diagnoses confirmed and recorded by licensed medical personnel were included. When data from multiple sources (e.g., clinical records, psychiatric reports) were integrated, trained medical staff independently extracted the data from each source, compared information across sources, and resolved any discrepancies through discussion, ensuring consistency and minimizing subjective bias.

### 2.4. Ethics Statement

The study was conducted in accordance with the Declaration of Helsinki and approved by the Institutional Ethics Committee of the University of Macerata (Protocol No. 100324). Written informed consent was obtained from all participants prior to their inclusion in the research. Participants received an information sheet outlining the study’s purpose and procedures and were informed that it was a descriptive study that did not pose any health risks. They were also informed of their right to withdraw from the study at any time without consequences. Confidentiality was ensured, and pseudonyms were used in reporting findings to protect participants’ identities.

### 2.5. Statistical Analysis

Descriptive statistics for the only continuous variable in the data set (i.e., age) are presented as mean ± standard deviation and as median and interquartile range (25th–75th percentile). For comparison purposes with the male resident population of the province of Frosinone (Lazio, Italy), age was then divided into six categories. For contextual interpretation, the age and nationality distribution of the inmate cohort was compared with that of the male resident population of the province of Frosinone, using publicly available demographic data from the same reference period [20].

This comparison made it possible to calculate the proportion of inmates per 100 inhabitants for each age group and nationality category, thus providing a population-based framework for interpreting the observed morbidity patterns. All other variables are given as absolute frequencies and percentages. Comparisons of disease prevalence between age strata and between Italian and foreign nationals, as well as the association between pharmacological treatment and some specific diagnoses, were performed using the Pearson χ^2^ test.

To account for the possible influence of age and nationality, multivariable logistic regression models were performed for each disease category and for multimorbidity. In each model, the dependent variable was the binary presence or absence of the disease, and the independent variables included age (continuous) and nationality (foreign vs. Italian as reference). The results of the regression analyses were expressed as odds ratios (ORs) with corresponding 95% confidence intervals (CIs). Multivariable logistic regression models were specified a priori to include age and nationality as potential confounders of the association between incarceration and health status. Univariable screening was not performed, as the selection of covariates was guided by expertise rather than statistical significance in bivariate analyses, in line with STROBE recommendations. Model fit and overall significance were evaluated using the likelihood ratio chi-square (LR^2^) test, comparing each full model with its intercept-only counterpart.

Smoking was also considered as a potential risk factor for the diseases studied, but its extremely high prevalence in the cohort studied (more than 99%) precluded an accurate estimation of its effects in the models. The OR for the association between drug dependence and psychiatric disorders was calculated directly from the 2 × 2 contingency table as the ratio between the probability of having a psychiatric disorder in the drug-dependent group and the probability in the non-drug-dependent group. Statistical significance was set at a two-sided *p*-value < 0.05. All analyses were performed using Stata version 19 (StataCorp., College Station, TX, USA).

## 3. Results

The study population consisted of 477 inmates, with a mean age of 47.3 ± 13.1 years (median 49; IQR 37–58). Of these, 332 (69.6%) were Italian nationals and 145 (30.4%) were foreign nationals, mainly from Eastern Europe and Africa. Compared to the male resident population of the province of Frosinone (n = 195,636), the prison cohort had a significantly different age distribution [20]. Inmates were overrepresented in the 35- to 69-year-old age group, with differences of +7.2 percentage points for the 35- to 44-year-old group, +3.3 for the 45- to 54-year-old group, +3.4 for the 55- to 64-year-old group and +7.0 for the 65- to 69-year-old group, while they were underrepresented in the 18- to 24-year-old (−5.2 percentage points) and ≥70-year-old (−18.4 percentage points) age groups. The proportion of inmates per 100 inhabitants reached its maximum in the 65–69 age group (0.47%), followed by the 35–44 age group (0.35%), the 45–54 age group (0.29%) and the 55–64 age group (0.29%), with the lowest proportion observed in the 70 and older age group (0.01%). Regarding the distribution of nationalities, foreign residents in the province accounted for only 9,798 people (5.0%) in the male resident population, but 145 people (30.4%) in the prison population [20]. This corresponds to a ratio of 1.48% for foreigners versus 0.18% for Italians, i.e., an 8.3-fold difference between inmates and residents. Of the 474 current smokers (99.4% of the total cohort), the majority were classified as heavy smokers (>20 cigarettes/day; n = 286, 60.4%), followed by moderate smokers (11–20 cigarettes/day; n = 185, 39.0%) and a very small proportion of light smokers (≤10 cigarettes/day; n = 3, 0.6%). The distribution suggests that virtually all smokers in the cohort had moderate to high daily tobacco consumption, limiting the possibility of meaningful subgroup comparisons and emphasizing the substantial tobacco exposure in this population. The full results are shown in Table 1.

In terms of morbidity, 265 inmates (55.6%) had at least one diagnosed pathology and 149 of them (56.2%) had two or more conditions, including 111 (41.9%) with exactly two, 32 (12.1%) with three, and 6 (2.3%) with four or five conditions. The most common diagnostic categories were psychiatric disorders, which occurred in 95 inmates (19.9%), and cardiovascular disorders, which occurred in 82 inmates (17.2%). Osteoarticular diseases affected 69 inmates (14.5%), endocrine–metabolic diseases 66 (13.8%), diseases of the oral cavity 59 (12.4%), and diseases of the respiratory tract 58 (12.2%). In the cardiovascular disease category, all 82 cases had arterial hypertension and 70 (85.4%) had ischemic heart disease. Among the psychiatric disorders, anxiety disorders accounted for 60 cases (63.2%), followed by psychotic disorders in 10 (10.5%), depressive disorders in 10 (10.5%), and borderline personality disorders in 10 (10.5%). In the group of endocrine–metabolic disorders, dyslipidemia was diagnosed in 37 cases (56.1%), type 2 diabetes in 17 cases (25.8%), and hypothyroidism in 16 cases (24.2%). In terms of respiratory diseases, COPD (Chronic Obstructive Pulmonary Disease) was diagnosed in 32 inmates (55.2%), chronic bronchitis in 14 (24.1%), asthmatic bronchitis in 9 (15.5%), and obstructive sleep apnea syndrome in 4 (6.9%). Infectious diseases were detected in 9 inmates (1.9%), including HIV in 5, hepatitis C virus in 3, and hepatitis B virus in 1. Benign prostatic hyperplasia was observed in 14 inmates (2.9%). Drug dependence was found in 41 inmates (8.6%), of whom 26 (63.4%) were receiving suboxone and 15 (36.6%) methadone. Two hundred and fifty-one inmates (52.6%) were receiving treatment. The most frequently reported pharmacologic treatments were psychotropic drugs (n = 120, 25.2%) and antihypertensive agents (n = 82, 17.2%), followed by NSAIDs (non-steroidal anti-inflammatory drugs, n = 69, 14.5%). Endocrine–metabolic therapies were less common, including insulin or oral hypoglycemic agents (n = 24, 5.0%) and thyroid hormone replacement therapy (n = 20, 4.2%). Bronchodilators and gastroprotective medications were prescribed to a minority of inmates (n = 9, 1.9% and n = 3, 0.6%, respectively). All patients with a diagnosis of cardiovascular disease received antihypertensive medication (*p* < 0.001). More than 79% of patients with psychiatric disorders were treated with psychotropic drugs (*p* < 0.001). The details of disease prevalence among inmates and pharmacologic treatments are shown in Table 2.

Among inmates with documented drug addiction (n = 41), 16 (39.0%) had a psychiatric disorder, a prevalence more than twice that observed in inmates without drug addiction (18.1%). The association was statistically significant (*p* = 0.001) and corresponded to an odds ratio of 2.89 (95% CI 1.48–5.67), indicating that inmates with drug addiction had nearly three times the odds of having a psychiatric disorder compared with those without addiction.

Analysis by age group revealed strong, statistically significant increases in the prevalence of cardiovascular disease, benign prostatic hyperplasia, and endocrine–metabolic, respiratory, and osteoarticular diseases, as well as multimorbidity, with the highest proportions consistently found in inmates aged 65–69 and ≥70 years (Table 3). Conversely, drug addiction was most prevalent among inmates aged 25–34 years and declined progressively with increasing age (*p* < 0.001). Oral cavity and digestive diseases also decreased with age, while the prevalence of psychiatric and infectious diseases did not differ significantly between age groups (*p* = 0.667 and *p* = 0.241, respectively).

In unadjusted comparisons between Italians and foreign nationals, the prevalence of cardiovascular disease was higher in Italians (68 cases, 20.5%) than in foreign nationals (14 cases, 9.7%; *p* = 0.004) (Table 4). Endocrine–metabolic diseases were present in 55 Italians (16.6%) and 11 foreign nationals (7.6%; *p* = 0.009), and respiratory diseases in 50 Italians (15.1%) compared with 8 foreign nationals (5.5%; *p* = 0.003). Conversely, oral cavity diseases were far more common in foreign nationals (43 cases, 29.7%) than in Italians (16 cases, 4.8%; *p* < 0.001), and drug addiction was also more frequent among foreign nationals (20 cases, 13.8%) than Italians (20 cases, 6.3%; *p* = 0.007). No significant crude differences were observed between the two groups for psychiatric disorders, infectious diseases, or multimorbidity.

Multivariable logistic regression confirmed age as the main determinant of chronic disease and multimorbidity. Each additional year of age was associated with a higher probability of cardiovascular disease (OR 1.11, 95% CI 1.07–1.14, *p* < 0.001), benign prostatic hyperplasia (OR 1.24, 95% CI 1.10–1.38, *p* < 0.001), endocrine–metabolic disease (OR 1.08, 95% CI 1.05–1.11, *p* < 0.001), respiratory diseases (OR 1.10, 95% CI 1.06–1.14, *p* < 0.001), osteoarticular diseases (OR 1.08, 95% CI 1.05–1.12, *p* < 0.001), and multimorbidity (OR 1.05, 95% CI 1.03–1.07, *p* < 0.001). Age was inversely associated with drug dependence (OR 0.89, 95% CI 0.85–0.92, *p* < 0.001) and diseases of the oral cavity (OR 0.94, 95% CI 0.92–0.97, *p* < 0.001). Foreign nationality remained strongly associated with oral cavity diseases (OR 4.96, 95% CI 2.59–9.52), osteoarticular disorders (OR 3.97, 95% CI 1.95–8.10), and multimorbidity (OR 2.14, 95% CI 1.15–3.99; all *p* < 0.01) (Table 5).

## 4. Discussion

This study provides original empirical data on the health status of the entire inmate population of a single Italian prison facility, using clinically verified diagnoses and standardized ICD-10-CM classifications. While the international literature on prison health is extensive, data from the Italian context remain limited, often based on administrative sources rather than detailed clinical records. Through the integration of multiple data sources, including medical records, specialist evaluations, and pharmacy registries, and the application of rigorous diagnostic criteria, this study offers a systematic and reproducible characterization of chronic, psychiatric, and infectious diseases within a real-world correctional setting. Compared with the general male population of the Frosinone province, inmates showed a disproportionate representation of middle-aged and older individuals, particularly in the 65–69 age group. Foreign nationals, mainly from Eastern Europe and Africa, were markedly overrepresented in the prison compared to the local male population, reflecting broader migration-related incarceration dynamics observed in Europe [21]. Regarding health status, cardiovascular diseases emerged as one of the most prevalent conditions (17.2% of the total cohort), with a marked age gradient and universal presence of arterial hypertension among affected cases. Prevalence was significantly higher among Italian inmates compared with foreign inmates and increased sharply in older age groups, reaching 100% in those aged ≥70 years. This burden is likely linked to the extremely high rate of smoking reported by inmates themselves (99.4%), predominantly at heavy consumption levels, and to limited access to preventive healthcare services [22,23]. Other factors, such as sedentary lifestyle, chronic stress, low life satisfaction, and conditions related to incarceration, significantly contribute to cardiovascular risk. Future studies may clarify whether incarceration itself could act as an independent risk factor for cardiovascular disease, given that weight gain and increases in BMI have been described in the early years of detention, potentially associated with physical inactivity, unhealthy diet, and high levels of psychological stress [24]. Psychiatric disorders were also highly represented (19.9%), with anxiety disorders accounting for over 60% of cases, followed by psychotic, depressive, and borderline personality disorders in equal proportions. More than 79% of inmates with psychiatric disorders received psychotropic medication, underscoring the centrality of pharmacological management in this context. The association between drug addiction and psychiatric morbidity was strong, with inmates affected by substance dependence exhibiting nearly triple the odds of having a psychiatric disorder compared to those without addiction. These results mirror national data on people in custody [13] and are almost double the prevalence observed in the general Italian population (~10%) [17]. Contributing factors include environmental stressors inherent to incarceration, chronic stress, social isolation, and insufficient mental health support [25,26]. Respiratory diseases (12.2%) and endocrine–metabolic conditions (13.8%) were also common, with COPD and dyslipidemia as the leading specific diagnoses. Both categories showed a marked association with age, in line with the progressive accumulation of multimorbidity in older inmates. Conversely, drug addiction and oral cavity diseases were more frequent among younger individuals and were strongly associated with foreign nationality. In particular, oral cavity diseases were nearly five times more prevalent among foreign inmates, suggesting possible inequalities in access to dental care before incarceration. The study also documented a relatively low prevalence of infectious diseases (HIV, HCV, HBV) compared with European data, and no tuberculosis cases. This may reflect targeted institutional policies, such as the transfer of infected individuals to specialized facilities, that could be effective in reducing transmission [27,28,29,30]. Although the facility examined has an overcrowding rate slightly below the national average, the high prevalence of chronic and psychiatric conditions demonstrates that significant health burdens persist even in prisons not experiencing maximal congestion. Beyond overcrowding, structural determinants such as the prison’s organizational model, staff-to-inmate ratios, and shortages in the health workforce may also influence disease prevalence. The limited availability of specialized services, fragmented continuity of care with community healthcare, and resource constraints can exacerbate chronic and psychiatric morbidity. These factors should be considered when interpreting the observed health burden. These findings suggest that overcrowding is not the sole determinant of health vulnerability; structural, behavioral, and socio-demographic factors also play crucial roles. Pharmacological treatments were consistent with diagnosed conditions, with 52.6% of inmates receiving therapy. Psychotropic medications (25.2%) and antihypertensive agents (17.2%) were the most frequently prescribed, reflecting the high burden of psychiatric and cardiovascular diseases. NSAIDs were also commonly used (14.5%), related to osteoarticular conditions. The widespread use of psychotropic and cardiovascular medications highlights the critical need for continuous, coordinated pharmacological management within correctional healthcare settings. In light of these results, a comprehensive review of prison healthcare in Italy is warranted. Priorities should include improved access to prevention and management strategies for chronic diseases [13], the implementation of smoking cessation programs, better nutrition, expansion of mental health services, and reinforcement of addiction treatment pathways [31]. Structural reforms to reduce overcrowding, such as shortening pre-trial detention, promoting alternative sentencing for non-violent offenders, and investing in community-based sanctions, may further improve health outcomes [11,32]. Our findings partially align with the WHO Regional Office for Europe’s 2022 Status Report, which identifies cardiovascular and psychiatric disorders as primary health burdens in European prisons. In contrast, the lower prevalence of communicable diseases observed in our cohort may reflect specific national or facility-level practices, such as the transfer of infected inmates [33]. This pattern is consistent with international evidence showing that incarceration entails both elevated health risks and unique opportunities for intervention [13,33,34]. In this regard, retrospective analyses of deaths in custody in Italy have highlighted cardiovascular disease as the leading cause of natural death, whereas suicide—often associated with psychiatric or substance use disorders—represents the most frequent violent cause [35,36,37]. This reinforces the urgent need for integrated psychiatric and addiction care, in compliance with human rights standards and the constitutional right to health. Overall, the results highlight the co-existence of high-risk behaviors (heavy smoking), chronic conditions, and psychiatric disorders in an aging inmate population, with significant disparities linked to nationality. Rather than proposing generic systemic reforms, these data support targeted facility-level interventions aimed at strengthening screening, ensuring treatment continuity, and promoting health literacy within prisons. Finally, prison health should be contextualized within a broader framework that considers the social and structural determinants of criminal behavior. Recent scholarship has suggested the possibility that chronic exposure to disadvantage and incarceration might exert influences at the biological level, with some authors hypothesizing mechanisms such as epigenetic changes. While these remain theoretical perspectives, they contribute to the broader discussion on the potential long-term impact of punitive versus rehabilitative models [38]. Environmental and social stressors may induce heritable molecular changes in gene expression without altering DNA sequences, potentially influencing health and behavioral outcomes across generations. While clinical and legal applications remain under study, this perspective encourages a justice model that not only treats individual offenders but also addresses the structural roots of criminality, aiming for both rehabilitation and prevention [38].

## 5. Limitations

This study has some limitations. Smoking habits were self-reported by inmates, which may introduce information bias due to underreporting or misclassification. The cohort included both sentenced and pre-trial detainees, but turnover data were not available, limiting the assessment of how population movement affects health outcomes. Additionally, data were collected exclusively from a male prison population, which restricts the generalizability of the findings to female or mixed-gender facilities. Furthermore, the study was conducted in a single institution, limiting broader applicability. Moreover, structural aspects such as the prison’s operational model, staff-to-inmate ratios, or health workforce shortages, which may influence the observed prevalence of disease, were not taken into account. These findings therefore refer to a single male prison facility and cannot be generalized to all Italian prisons, particularly female or mixed-gender institutions. Future research should incorporate objective smoking assessments and turnover data and include diverse penitentiary settings to improve generalizability.

## 6. Conclusions

This study highlights the substantial burden of psychiatric and chronic illnesses in a male prison population in Central Italy, particularly anxiety disorders, arterial hypertension, and self-reported heavy smoking. These findings underscore the need to strengthen mental health support, ensure access to specialized care, and implement targeted smoking cessation programs, all within a framework that respects the dignity and constitutional health rights of detainees. Integrated care pathways, coordinated by prison health services, regional authorities, and multidisciplinary teams, can facilitate systematic screening, continuity of treatment, and health promotion. Emphasis on modifiable risk factors such as tobacco use, nutrition, and physical activity is essential to mitigate long-term health risks. Facility-level interventions and structured cooperation with community healthcare services are key to improving health outcomes during incarceration and post-release. These findings support evidence-based, context-specific policies that safeguard inmate health while promoting rehabilitation and social reintegration.

## Figures and Tables

**Table 1:** Characteristics of the inmate population and comparison with male residents in the province of Frosinone.

	Inmates	Male Residents in Frosinone Province	% Inmates
N	477	195,636	0.24%
Age *	47.3 (13.1)		
18–24	16 (3.4)	16,784 (8.6)	0.10%
25–34	79 (16.6)	27,069 (13.8)	0.29%
35–44	110 (23.1)	31,022 (15.9)	0.35%
45–54	102 (21.4)	35,454 (18.1)	0.29%
55–64	98 (20.5)	33,543 (17.1)	0.29%
65–69	70 (14.7)	15,040 (7.7)	0.47%
70+	2 (0.4)	36,724 (18.8)	0.01%
Nationality, n (%)			
Italian	332 (69.6)	185,838 (95.0)	0.18%
Foreign	145 (30.4)	9798 (5.0)	1.48%
Ethnicity, n (%)			
African	64 (44.1)	N.A.	-
Asian	6 (4.1)	N.A.	-
Eastern European	69 (47.6)	N.A.	-
South American	6 (4.1)	N.A.	-
Smoker, n (%)	474 (99.4)	N.A.	-
Smoking categories (cigarette/days), n (%)			
Light smoker (≤10 cigarettes/days)	3 (0.6)	N.A.	-
Moderate smoker (11–20 cigarettes/days)	185 (39.0)	N.A.	-
Heavy smoker (>20 cigarettes/days)	286 (60.3)	N.A.	-

* Age is presented as mean ± standard deviation (median, 25th–75th). The other variables, included age range, are reported as absolute frequencies and (percentages). N.A.: not available.

**Table 2:** Prevalence of diseases in the inmate population, stratified by major diagnostic categories and specific conditions. Data are expressed as n (%).

	N (%)
Subjects with at least one pathology	265 (55.6)
One pathology	116 (43.8)
More than one pathology (≥2)	149 (56.2)
Cardiovascular Diseases	82 (17.2)
Arterial Hypertension	82 (100)
Ischemic Heart Disease	70 (85.4)
Benign Prostatic Hyperplasia	14 (2.9)
Endocrine–Metabolic Diseases	66 (13.8)
Hypothyroidism	16 (24.2)
Dyslipidemia	37 (56.1)
Type II Diabetes	17 (25.8)
Type I Diabetes	7 (10.6)
Respiratory System Diseases	58 (12.2)
Obstructive Sleep Apnea Syndrome (OSAS)	4 (6.9)
Chronic Obstructive Pulmonary Disease (COPD)	32 (55.2)
Chronic Bronchitis	14 (24.1)
Asthmatic Bronchitis	9 (15.5)
Infectious Diseases	9 (1.9)
HIV	5 (55.6)
HCV	3 (33.3)
HBV	1 (11.1)
Digestive System Disorders	7 (1.5)
Ulcerative Colitis	3 (42.9)
Crohn’s Disease	1 (14.3)
Erosive Gastritis	3 (42.9)
Oral Cavity Diseases	59 (12.4)
Psychiatric Disorders	95 (19.9)
Psychotic Disorder	10 (10.5)
Depressive Disorder	10 (10.5)
Anxiety Disorder	60 (63.2)
Borderline Personality Disorder	10 (10.5)
Substance Use Disorder	5 (5.3)
Osteoarticular Diseases	69 (14.5)
Drug Addiction	41 (8.6)
Suboxone	26 (63.4)
Methadone	15 (36.6)
Pharmacological treatments	
Antihypertensive drugs	82 (17.2)
Thyroid hormone replacement therapy	20 (4.2)
Insulin or oral hypoglycemic agents	24 (5.0)
Bronchodilators	9 (1.9)
Gastroprotective drugs	3 (0.6)
Psychotropic drugs	120 (25.2)
NSAIDs	69 (14.5)

**Table 3:** Distribution of diseases according to age groups (years) among inmates. Data are reported as n (%); *p*-values from chi-square tests.

	18–24 (n = 16)	25–34 (n = 79)	35–44 (n = 110)	45–54 (n = 102)	55–64 (n = 98)	65–69 (n = 70)	70+ (n = 2)	*p*-Value
Multiple Pathologies *	2/3 (66.7)	18/40 (45.0)	24/55 (43.6)	21/48 (43.8)	42/67 (62.7)	40/50 (80.0)	2/2 (100.0)	<0.001
Cardiovascular Disease	0 (0.0)	3 (3.8)	5 (4.5)	15 (14.7)	28 (28.6)	29 (41.4)	2 (100.0)	<0.001
Benign Prostatic Hyperplasia	0 (0.0)	0 (0.0)	0 (0.0)	0 (0.0)	6 (6.1)	7 (10.0)	1 (50.0)	<0.001
Endocrine–Metabolic Disease	0 (0.0)	6 (7.6)	6 (5.5)	9 (8.8)	19 (19.4)	24 (34.3)	2 (100.0)	<0.001
Respiratory System Disease	0 (0.0)	5 (6.3)	2 (1.8)	8 (7.8)	15 (15.3)	26 (37.1)	2 (100.0)	<0.001
Infectious Diseases	0 (0.0)	2 (2.5)	5 (4.5)	2 (2.0)	0 (0.0)	0 (0.0)	0 (0.0)	0.241
Digestive System Disorders	1 (6.3)	4 (5.1)	1 (0.9)	1 (1.0)	0 (0.0)	0 (0.0)	0 (0.0)	0.051
Oral Cavity Diseases	0 (0.0)	1 (25.0)	1 (100.0)	1 (100.0)	0 (0.0)	0 (0.0)	0 (0.0)	<0.001
Psychiatric Disorders	3 (18.8)	17 (21.5)	27 (24.5)	20 (19.6)	14 (14.3)	14 (20.0)	0 (0.0)	0.667
Osteoarticular Diseases	0 (0.0)	3 (3.8)	11 (10.0)	9 (8.8)	35 (35.7)	10 (14.3)	1 (50.0)	<0.001
Drug Addiction	3 (18.8)	24 (30.4)	11 (10.0)	1 (1.0)	2 (2.0)	0 (0.0)	0 (0.0)	<0.001

* The percentage was determined considering the number of subjects with at least one pathology as the denominator.

**Table 4:** Distribution of diseases by origin (Italian vs. foreign inmates). Data are reported as n (%); *p*-values from chi-square tests.

	Italy (n = 332)	Foreign (n = 145)	*p*-Value
Multiple Pathologies *	101/183 (55.2)	48/82 (58.5)	0.612
Cardiovascular Disease	68 (20.5)	14 (9.7)	0.004
Benign Prostatic Hyperplasia	12 (3.6)	2 (1.4)	0.183
Endocrine–Metabolic Disease	55 (16.6)	11 (7.6)	0.009
Respiratory System Disease	50 (15.1)	8 (5.5)	0.003
Infectious Diseases	4 (1.2)	5 (3.4)	0.098
Digestive System Disorders	4 (1.2)	3 (2.1)	0.470
Oral Cavity Diseases	16 (4.8)	43 (29.7)	<0.001
Psychiatric Disorders	68 (20.5)	27 (18.6)	0.640
Osteoarticular Diseases	45 (13.6)	24 (16.6)	0.392
Drug Addiction	21 (6.3)	20 (13.8)	0.007

* The percentage was determined considering the number of subjects with at least one pathology as the denominator.

**Table 5:** Multivariate logistic regression models assessing the association of age (continuous) and origin (reference: Italy) with the presence of selected diseases in the inmate population.

	CVD	BPH	EMD	RSD	OCD	PD	OAD	DA	MP
Age	1.11 *** (1.07–1.14)	1.24 *** (1.10–1.38)	1.08 *** (1.05–1.11)	1.10 *** (1.06–1.14)	0.94 *** (0.92–0.97)	0.99 (0.97–1.00)	1.08 *** (1.05–1.12)	0.89 *** (0.85–0.92)	1.05 *** (1.03–1.07)
Nationality §	1.50 (0.72–3.18)	5.39 (0.73–39.61)	1.07 (0.49–2.35)	1.10 (0.45–2.70)	4.96 *** (2.59–9.52)	0.75 (0.43–1.29)	3.97 *** (1.95–8.10)	0.90 (0.44–1.85)	2.14 ** (1.15–3.99)
N	477	477	477	477	477	477	477	477	265
LR chi2	72.92	30.61	40.05	51.70	68.16	2.33	39.72	60.69	18.60
*p*-value	<0.001	<0.001	<0.001	<0.001	<0.001	0.3118	<0.001	<0.001	<0.001

Results are expressed as odds ratios (OR) with 95% confidence intervals (CI). *p* < 0.05 was considered statistically significant (** *p* < 0.01; *** *p* < 0.001). § Reference: Italian nationality. Abbreviations: CVD, Cardiovascular Disease; BPH, Benign Prostatic Hyperplasia; EMD, Endocrine–Metabolic Disease; RSD, Respiratory System Disease; OCD, Oral Cavity Diseases; PD, Psychiatric Disorders; OAD, Osteoarticular Diseases; DA, Drug Addiction; MP, Multiple Pathologies.

## Data Availability

The data presented in this study are not publicly available due to privacy and ethical restrictions related to the protection of sensitive personal and health information of the individuals involved.
